# Facile Cu–MOF-derived Co_3_O_4_ mesoporous-structure as a cooperative catalyst for the reduction nitroarenes and dyes

**DOI:** 10.1038/s41598-024-52708-x

**Published:** 2024-03-21

**Authors:** Masoume Malmir, Majid M. Heravi, Elham Shafiei Toran Poshti

**Affiliations:** Department of Organic Chemistry, Faculty of Chemistry, Alzahra University, PO Box: 1993891176, Tehran, Iran

**Keywords:** Chemistry, Catalysis, Heterogeneous catalysis

## Abstract

The present study describes the environmentally friendly and cost-effective synthesis of magnetic, mesoporous structure-Co_3_O_4_ nanoparticles (m-Co_3_O_4_) utilizing almond peel as a biotemplate. This straightforward method yields a material with high surface area, as confirmed by various characterization techniques. Subsequently, the utilization of m-Co_3_O_4_, graphene oxide (GO), Cu(II)acetate (Cu), and asparagine enabled the successful synthesis of a novel magnetic MOF, namely GO–Cu–ASP–m-Co_3_O_4_ MOF. This catalyst revealed remarkable stability that could be easily recovered using a magnet for consecutive use without any significant decline in activity for eight cycles in nitro compound reduction and organic dye degradation reactions. Consequently, GO–Cu–ASP-m-Co_3_O_4_ MOF holds immense potential as a catalyst for reduction reactions, particularly in the production of valuable amines with high industrial value, as well as for the elimination of toxic-water pollutants such as organic dyes.

## Introduction

The issue of ecological pollution resulting from the rapid advancement of technology in the industry has gained momentous attention in recent years^1–5^. Nitrophenols, as an example, are crucial industrial compounds; however, their presence as water pollutants can pose severe harm and toxicity to human and aquatic life. These compounds can be transformed into aminophenols through chemical reduction, presenting various alternative applications^6–8^. Aromatic amines, in general, are relatively safer chemicals with diverse biological and synthetic uses, including photographic improvement, synthesis of dye intermediate, optical brighteners, corrosion inhibition, anti-corrosion lubrication, agricultural chemicals, and pharmaceuticals for the drug's production^9–11^. The reduction of nitroarenes and dyes is a primary and important transformation with industrial implications in sectors such as textiles, paper, food, and pharmaceuticals^12–14^. Numerous approaches, including oxidation and degradation, have been employed to mitigate or eliminate the 4-nitrophenol (4-NP) in the environment^15–18^. Conversely, these approaches are costly and also have demonstrated only partial effectiveness. In contrast, the utilization of sodium borohydride as a low-cost technique is widely employed in numerous cleaning processes.

Dyes are extensively employed in productions and chemical industries such as food, pharmaceuticals, textiles, paper, and among others^12,14^. However, their excessive use has resulted in environmental pollution due to their undesirability, high visibility, and resistance to degradation, and contamination of wastewater. Therefore, it is crucial to control industrial effluents to ensure a fresh and harmless environment. Congored (CR) and Methylene blue (MB) are commonly used anionic and cationic dyes^19^, which can cause significant ecological harm if discharged without suitable treatment. Consequently, improvement of a unique and efficient way for degradation of dye has become increasingly important. In this regard, metal nanoparticles display superior catalytic performance in the degradation of organic dyes^20^.

Heterogeneous catalysis offers numerous advantages over homogeneous catalysis, including high catalytic activity and high selectivity resulting from a large surface area, excellent stability and reusability^21^. Nanoparticle-based heterogeneous catalysts are particularly intriguing because of their surface area properties and catalytic activity by adjusting their shapes. While noble metals have demonstrated superior catalytic activities, their usage is narrow due to the high cost^22^. In its place, transition metal-catalysts have been widely employed to overcome these limitations. These catalysts were utilized in various transformations, such as oxidations^23–27^, degradation of eosin Y and MB^28^, 2,4-dimethyl phenol^29^, Orange (II)^30^, crystal violet dye^31^ among other examples.

On the other hand, nanomagnetic catalysts are highly attractive because of their ease of isolation and reusability. Various nanoparticles with magnetic properties were broadly synthesized and utilized in wide range of applications^32–35^. Co_3_O_4_ is an encouraging material due to its environmental benignity, excellent activity, low cost, and morphology-dependent properties^36^. In the past, the synthesis of Co_3_O_4_ has involved hazardous solvents or complicated processes. However, recent advancements have enabled the synthesis of Co_3_O_4_ using biotemplates instead of harsh chemicals^37,38^. Additionally, pollen-assisted CeO_2_/Co_3_O_4_ hollow microspheres have been developed for photocatalytic applications^39^.

Graphene oxide (GO) is a promising candidate as a support for improving the catalytic performance of transition-metal catalysts due to its excellent surface's properties^40^. GO has been positively operated as a support for dispersing as well as stabilizing metal nanoparticles^41^. Due to the attractive dispersion of GO in aqueous solution, is known as a support for the fabrication of water-soluble nanocarbon.

On the other wise, another intriguing nanomaterial with inherent properties such as high stability, strength in harsh environments, and facile accessibility is the porous metal–organic-framework (MOF). MOFs consist of inorganic metal ions and organic linkers or ligands, offering a wide range of tunable properties including structure, density, and surface area^42,43^. These MOFs find applications in gas storage, drug delivery, and catalytic activity^44–49^. The field of MOF composites has witnessed significant expansion in recent years^50–59^, particularly in fuel cells, photocatalysis, oxygen reduction, and other catalytic applications^60^. Heterogeneous MOFs^61–63^, especially those incorporating magnetic nanoparticles, have gained popularity due to their favorable separation characteristics, preventing aggregation and preserving the unique properties of nanoscale materials^64^.

Motivated by these considerations and our ongoing research in heterogeneous catalysts field^65–74^, we current our findings on the decoration and synthesis of a new magnetic MOF-based catalyst containing m-Co_3_O_4_. This catalyst is fabricated through a stepwise functionalization process, where graphene oxide (GO) is functionalized with Cu(OAc)_2_ and asparagine (ASP), and then coordinated with m-Co_3_O_4_, resulting in the creation of the porous magnetic MOF structure. Notably, we successfully utilize almond peel as a template to synthesize porous Co_3_O_4_ nanoparticles with magnetic properties. Almond shell as a natural, hydrophilic and cost-effective template composed of hemicelluloses, lignin and cellulose^75^, plays a fundamental role in determining the morphology and surface area of prepared m-Co_3_O_4_ nanoparticles. The catalyst structure is confirmed by various standard techniques, and its performance towards the nitroarenes, MB and CR reduction under ambient reaction conditions is assessed. This result demonstrate that GO–Cu–ASP–m-Co_3_O_4_ MOF as a catalyst displays promising catalytic behavior, surpassing that of other catalysts reported in this field. Moreover, it has the potential to enhance the durability of various catalytic progressions.

## Results and discussion

### Catalyst characterization

After successful completion of catalyst synthesis, various techniques were employed to identify the catalyst. Initially, the stepwise synthesis of GO–Cu–ASP–m-Co_3_O_4_ MOF, including GO (A), GO–Cu (B), GO–Cu–ASP (C), Almond peel (D), Co_3_O_4_ (E) and GO–Cu–ASP–m-Co_3_O_4_ MOF (F) were investigated and identified using FTIR analysis (Figure S1). The absorption bands at 1050, 1222, 1614, 1728 and 3444 cm^−1^ in Figure S1A correspond to C–O, C–OH, C=C, C=O, and O–H groups in the structure of GO. In Figure S1B, a slight redshift in the absorption bands in 1200–1600 cm^−1^ indicates the loading of Cu particles onto the GO sheets. The significant absorption band at 1658 cm^−1^ in Figure S1C confirms the presence of an –CONH_2_ group and its interaction with GO–Cu. The absorption bands observed at 1622, 1735, 2927 and 3427 cm^−1^ in Figure S1D resemble to stretching vibrations of the C=C, C=O, O–H, and N–H, respectively, indicating the presence of almond peel. Additionally, two distinctive absorption bands at 567 and 634 cm^−1^ in Figure S1E provided evidence for the successful synthesis of magnetic m-Co_3_O_4_ (Figure S1E). The final catalyst's FTIR spectrum (Figure S1F) confirms the presence of all the absorption bands from the previous stages, albeit with slightly reduced peak intensities, indicating the stepwise incorporation of each stage into the magnetic catalyst GO–Cu–ASP–m-Co_3_O_4_ MOF.

**Figure 1:**
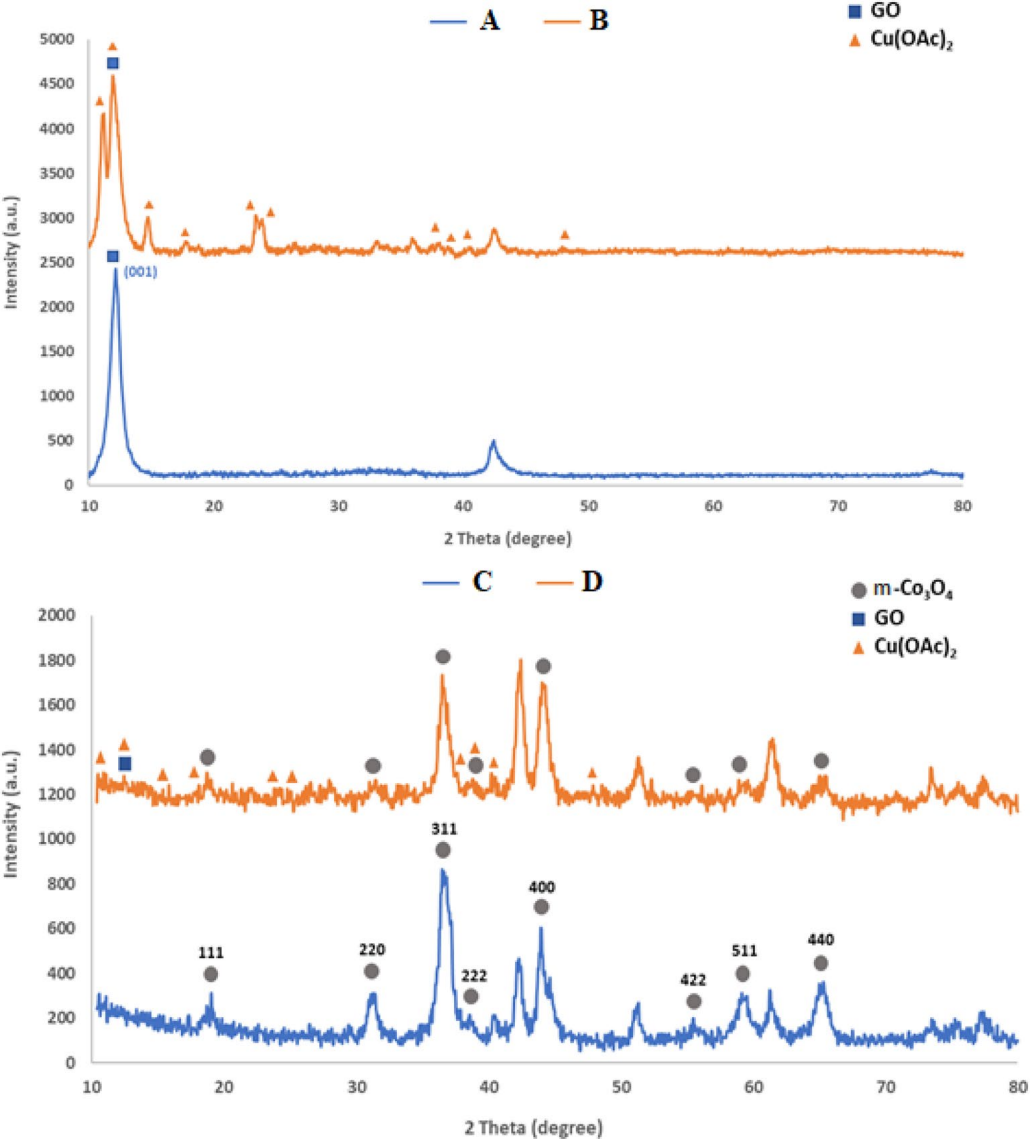
XRD patterns of (**A**) GO, (**B**) GO–Cu, (**C**) m-Co_3_O_4_, (**D**) GO–Cu–ASP–m-Co_3_O_4_ MOF.

The XRD patterns of GO, GO–Cu, m-Co_3_O_4_, GO–Cu–ASP–m-Co_3_O_4_ MOF and their comparison are depicted in Fig. 1. The distinct absorption band at 2*θ* = 11° (001), as indicated in Fig. 1A, is consistent with previous studies (JCPDS No. 89-8490) and approves the presence of a GO crystal^76^. In Fig. 1B, a sharp peak at 2*θ* = 11° (001), corresponding to GO, and anther peaks at 2*θ* = 11.56°, 14.5°, 16.7°, 22.78°, and 24.3°, attributed to Cu(OAc)_2_, provide confirmation of the synthesis of GO–Cu (JCDD No. 01-077-7718)^77^. In the spectrum 1C and in agreement with the previous literature^78^, the distinct sharp absorption peaks at 2*θ* = 19.0° (111), 31.6° (220), 36.9° (311), 38.7° (222), 45.2° (400), 56.1° (422), 59.8° (511), and 65.7° (440), correspond to the successful synthesis of m-Co_3_O_4_ (JCPDS No. 42-1467). Furthermore, distinct absorption peaks at 2*θ* = 11.56°, 15.56°, 16.54°, 25.74°, and 26.22° were observed, consistent with recent reports (JCDD No. 01/077-77), confirming the presence of a Cu-MOF (Fig. 1D)^77^. It should be noted that the presence of m-Co_3_O_4_ in the GO–Cu–ASP–m-Co_3_O_4_ MOF was also confirmed by observing these bands.

The Raman spectra of m-Co_3_O_4_ and GO–Cu–ASP–m-Co_3_O_4_ MOF are depicted in Figure S2, revealing two distinct absorption bands at 1335 cm^−1^ (D band associated with sp^3^ configuration) and 1570 cm^−1^ (G band attributed to graphitic carbon) for catalyst. As observed in Figure S2, D and G bands are fully preserved in the final catalyst, providing evidence for the successful synthesis of GO–Cu–ASP–m-Co_3_O_4_ MOF. Additionally, the I_D_/I_G_ ratio of 0.85 signifies a carbonaceous catalyst structure with some degree of disorder. It is noteworthy that m-Co_3_O_4_ exhibits several active Raman modes at ∼185, 465, 507, 604, 680, and 750 cm^−1^. Excluding the last mode, all observed modes are in agreement with the values of pure Co_3_O_4_ spinel structure^79^ yet with an average shift to right after functionalization with some reagents. While the Raman mode at 680 cm^−1^ is attributed to characteristics of the octahedral sites A_1_g, the Eg, and F_2_g modes are related to the combined vibrations of tetrahedral site and octahedral oxygen motions. Moreover, the average shift may be attributed to size effects or surface stress/strain.

The thermogravimetric analysis of GO–Cu (A), m-Co_3_O_4_ (B), and GO–Cu–ASP–m-Co_3_O_4_ MOF (C) was conducted at 25–700 °C, N_2_ atmosphere. Figure S3A depicts the TGA curve of GO–Cu, which exhibits two distinct decomposition stages within 25–700 °C. The first loss at 130 °C can be attributed to removal of hydroxyl groups and water. Subsequently, a second significant decomposition stage occurs between 190 and 400 °C, corresponding to the degradation of carboxylic groups and the release of CO_2_ gas. As shown in Figure S3B, corresponding to m-Co_3_O_4_, a satisfactory level of thermal stability was observed with minimal degradation. In the TG curve of GO–Cu–ASP–m-Co_3_O_4_ MOF, in addition to water and moisture loss, a major degradation event was observed within 230 to 450 °C, indicating better thermal stability compared to GO–Cu. GO–Cu–ASP–m-Co_3_O_4_ MOF undergoes decomposition with increasing temperature, releasing N_2_ and CO_2_ gases from its molecular structure.

**Figure 2:**
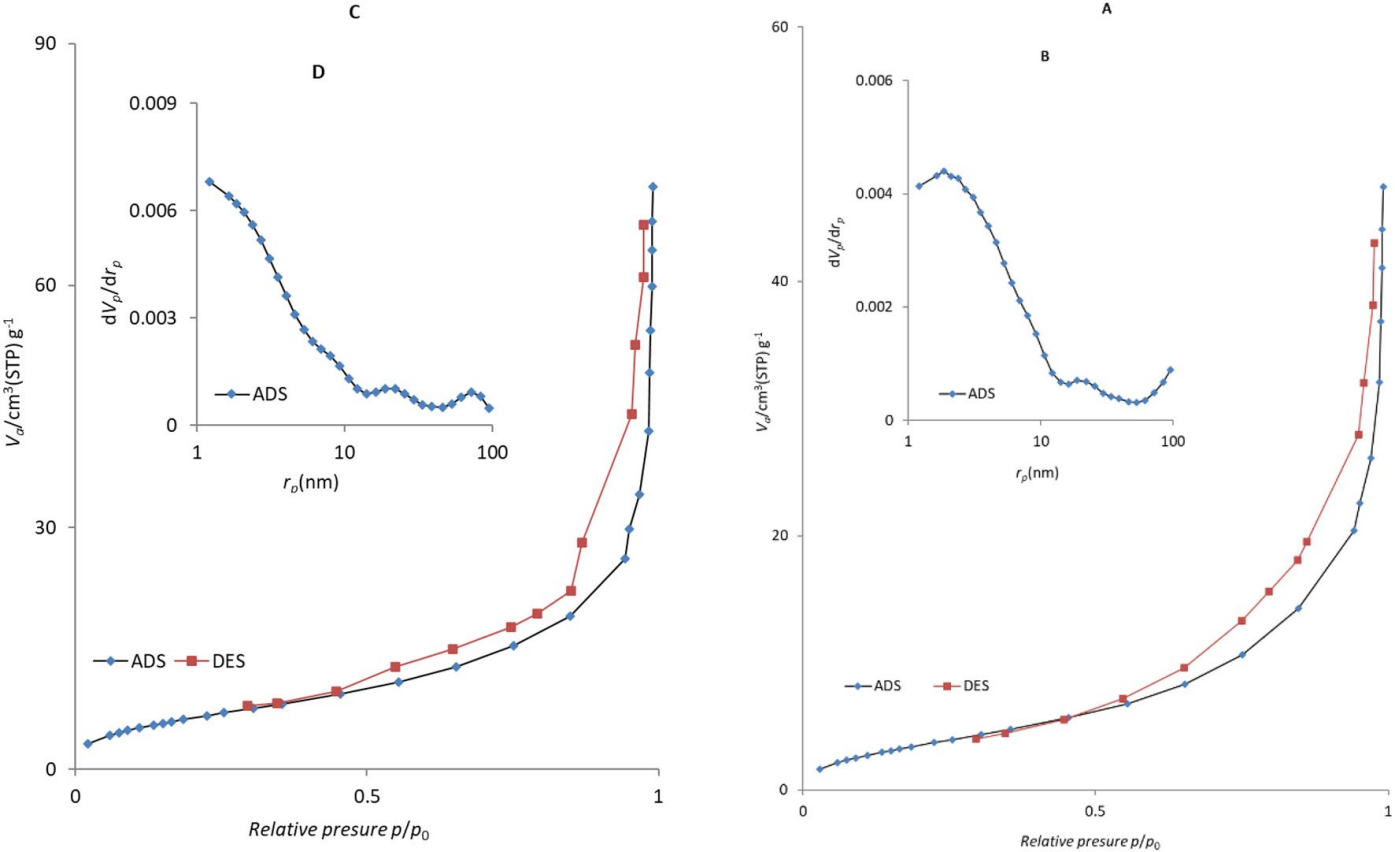
The N_2_ adsorption–desorption isotherms (**A**,**C**) and BJM-Plots (**B**,**D**) of the m-Co_3_O_4_ and GO–Cu–ASP–m-Co_3_O_4_ MOF.

To assess the structural characteristics of m-Co_3_O_4_ and GO–Cu–ASP–m-Co_3_O_4_ MOF, BET analysis was employed. Both isotherms displayed type II behavior with H3 hysteresis loops, demonstrating the porous structure that is retained after functionalization (Fig. 2A,C). The specific surface area, total pore volume, and average pore diameter of m-Co_3_O_4_ were determined as 14.11 m^2^g^−1^, 0.0693 cm^3^g^−1^ 19.65 nm, respectively. For GO–Cu–ASP–m-Co_3_O_4_ MOF, two values increased to 24.13 m^2^g^−1^, 0.108 cm^3^g^−1^ and pore diameter decreased to 17.90 nm. The heightened specific surface area and overall pore volume following the functionalization of GO–Cu indicate the successful incorporation of metal particles and the establishment of interconnections between the two porous structures. In agreement with BJH isotherms (Fig. 2B,D), m-Co_3_O_4_ exhibits two types of pores: micropores and mesopores with sizes of 3.5 and 25 nm, respectively, which after functionalization and formation of MOF, two mesoporous pores with approximate sizes of 35 and 90 nm appeared. Notably, these results consistent with HRTEM measurements and particle size analysis (Cu, m-Co_3_O_4_).

The saturation magnetization of GO–Cu–ASP–m-Co_3_O_4_ MOF catalyst was measured by VSM analysis and compared to bare m-Co_3_O_4_ (Figure S4). The saturation magnetization of m-Co_3_O_4_ was measured as 23.85 emu g^−1^. However, after multiple instances of doping and surface modification, this value decreased to 11.98 emu g^−1^. Nevertheless, the final catalyst still possessed favorable magnetic properties, enabling its effortless separation from the mixture by applying a magnet.

SEM/EDX analysis was accompanied to probe the morphology of Almond peel, m-Co_3_O_4_, GO–Cu–ASP–m-Co_3_O_4_ MOF, GO–Cu and GO–Cu–ASP. In Figure S5A, almond peel possesses a honeycomb-like structure with distinct layered features, and it exhibits a highly mesoporous nature. Based on the EDX result (Figure S5B), the almond peel contains various elements, including carbon (56.55%), oxygen (34.83%), calcium (3.27%), silica (0.57%) and nitrogen (4.82%). In Figure S5C, SEM image displays a cavity-like morphology (predominantly) and rod-like structures of Co_3_O_4_ (C). As shown in Figure S5D, EDX analysis of Co_3_O_4_ confirms its elemental composition, consisting of carbon (5.45%), nitrogen (0.52%), oxygen (2.99%), calcium (0.47%), and cobalt (90.5%). These observations provide evidence of the successful synthesis of Co_3_O_4_. Furthermore, Figure S5E and F depict the images of GO–Cu and GO–Cu–ASP, respectively. GO–Cu represents GO sheets, while GO–Cu-ASP corresponds to a specific form of GO–Cu modified with asparagine. Figure S5G and H reveal the final structure of the catalyst, which consists of a combination of m-Co_3_O_4_ and GO–Cu–ASP. Overall, GO–Cu–ASP–m-Co_3_O_4_ MOF exhibits well-defined cavity, layered, and rod-like structures in some parts. The EDX analysis of GO–Cu–ASP–m-Co_3_O_4_ MOF indicates the presence of carbon (12.01%), nitrogen (2.01%), oxygen (4.68%), silica (0.1%), calcium (0.24%), cobalt (80.57%), and copper (0.39%), confirming the correct synthesis of the catalyst. The results obtained from the EDX analysis were consistent with those obtained from ICP-OES. The Cu and Co loadings were determined to be 0.0028 mmol g^−1^ and 0.11 mmol g^−1^, respectively. Additionally, the amounts of Cu and Co leaching after las recycle were calculated as 0.0024 mmol g^−1^ and 0.086 mmol g^−1^, respectively.

**Figure 3:**
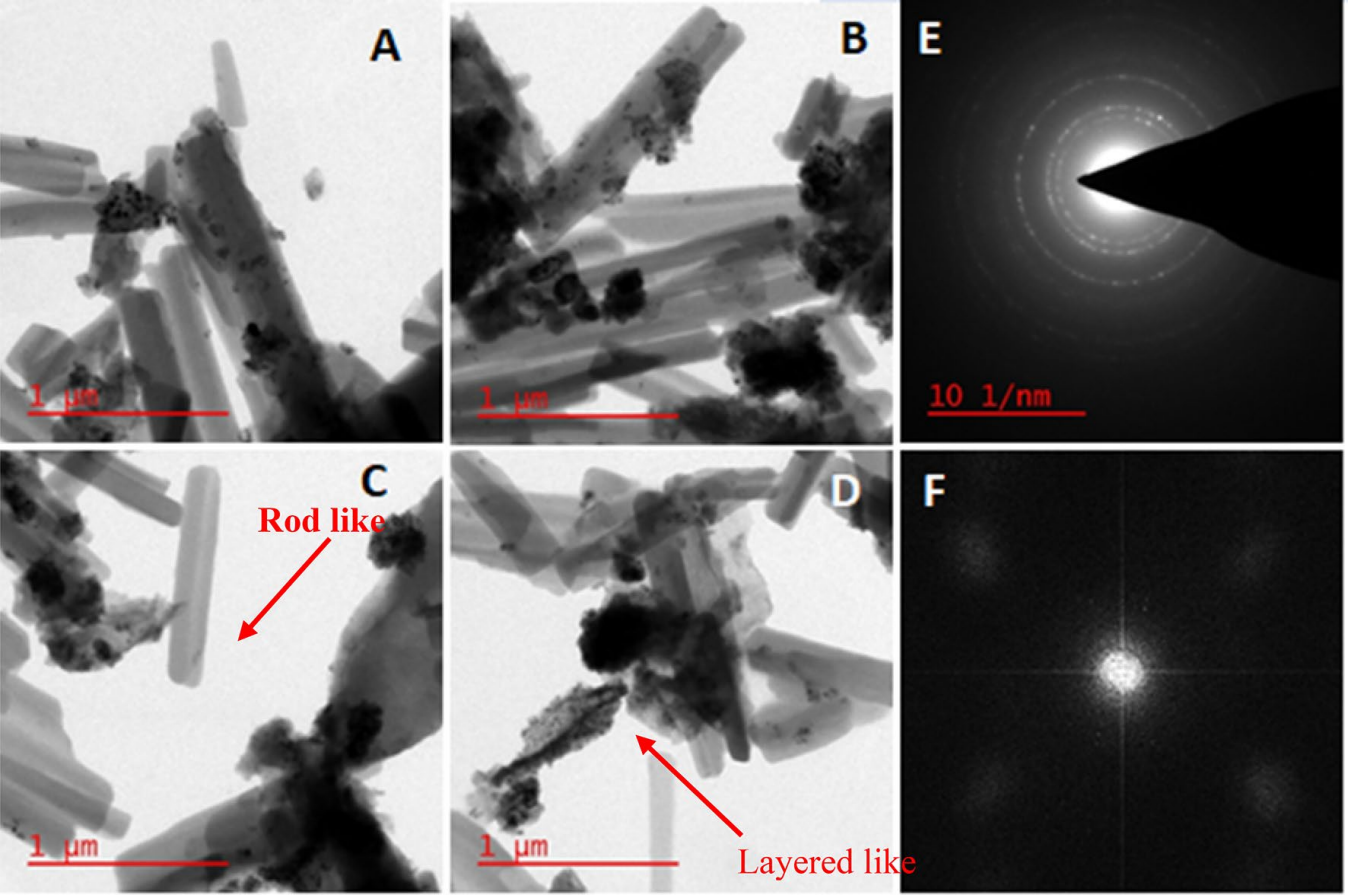
HRTEM images (**A**–**D**) and SAED pattern (**E** and **F**) of GO–Cu–ASP–m-Co_3_O_4_ MOF.

As illustrated in Fig. 3, the images acquired from HRTEM analysis vividly depict the thin layers of GO containing dark-colored spherical particles that remain intact after surface modification and catalyst structure refinement. Moreover, GO layer containing Cu species, exhibiting substantial interaction with the elongated m-Co_3_O_4_ nano rods and finely shaped copper nanoparticles. Figure 3A–D exhibit the significant interaction of GO–Cu–ASP sheets with long m-Co_3_O_4_ nano rods. The MOF structure is well-formed by these constituents. Cu presence, characterized by small particle sizes, is distinctly observable according to the SAED image. The SAED pattern confirms the presence of it and reveals size ranging from 20 to 55 nm (Fig. 3F).

### Investigation of the catalytic activity

**Figure 4:**
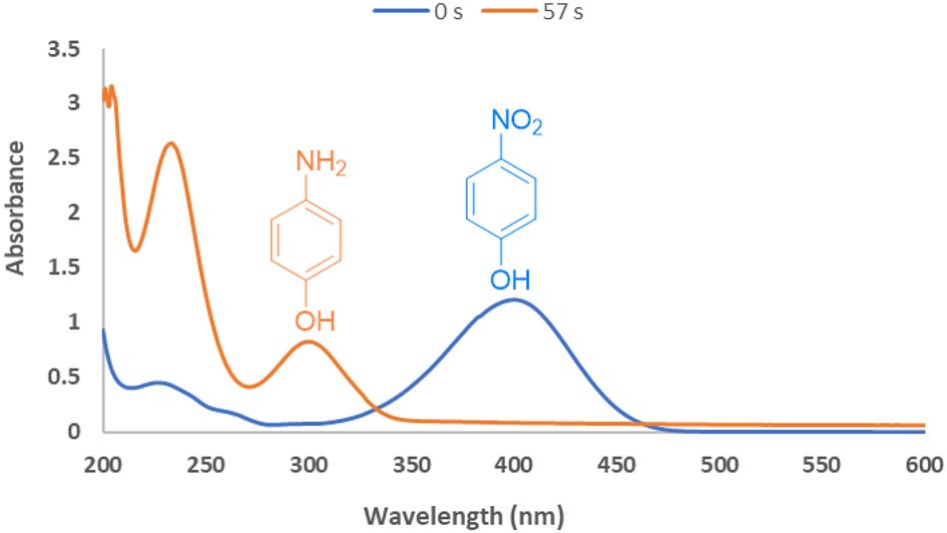
UV–Vis spectra of 4-NP reduction to 4-AP over GO–Cu–ASP–m-Co_3_O_4_ MOF under optimized conditions.

To assess the performance of GO–Cu–ASP–m-Co_3_O_4_ MOF catalyst, the reduction of 4-nitrophenol (4-NP) was nominated as a model reaction. The reaction conditions were optimized by varying the catalyst dosage, NaBH_4_ concentration, water content and prospering of active site (refer to Table S1 and Figure S6 for details). The presence of GO–Cu–ASP–m-Co_3_O_4_ MOF proved to be essential for the reduction reaction, as no reduction activity was detected without any catalysts (Table S1, entry 11). Based on the optimization of catalyst amounts, 30 mg of GO–Cu–ASP–m-Co_3_O_4_ MOF was selected for further experiments due to its high conversion rate (Table S1, entries 4–10). The use of very high or low amounts of GO–Cu–ASP–m-Co_3_O_4_ MOF was found to be less suitable, as it led to longer reaction times despite complete conversion. The effect of NaBH_4_ content (0, 5, 7.5, and 10 mmol) on the reduction process was investigated. Table S1 shows that the highest efficiency and absence of side products were achieved when 7.5 mmol NaBH_4_ was used. Increasing the amount of water had a significant impact on the reduction reaction time. Furthermore, the reduction process did not ensue in the absence of GO–Cu–ASP–m-Co_3_O_4_ MOF or without a base. The optimized conditions for the reduction of 4-NP were determined to be the use of 30 mg of GO–Cu–ASP–m-Co_3_O_4_ MOF with 7.5 mmol NaBH_4_ in 5 mL of H_2_O at room temperature, resulting in a 100% yield of 4-aminophenol (4-AP) in just 57 s. The progress of the reaction was monitored using TLC and UV–Vis analysis. TLC analysis with a polar solvent mixture of ethyl acetate and n-hexane in a 6:4 ratio confirmed the formation of 4-AP. UV–Vis analysis was also employed to ensure confidence in the reaction progress. Figure 4 illustrates a decrease in the absorption peak at 313 nm (indicating a yellow solution) for 4-NP, and the emergence of a strong peak at 400 nm (indicating a colorless solution) corresponding to nitrophenolate, confirming successful reduction in the presence of the catalyst. Upon the addition of GO–Cu–ASP–m-Co_3_O_4_ MOF, the absorption band observed at 400 nm was attenuated, and a new band appeared at 300 nm, indicating the formation of the 4-AP product. The lack of change in the intensity of this peak over time confirms that the reduction is not possible without a catalyst.

Following optimization of the reaction conditions, the GO–Cu–ASP–m-Co_3_O_4_ MOF was employed for the reduction of nitroaromatic compounds. As presented in Table 1 and Figure S7, the catalyst exhibited high reactivity and efficiency in reducing a wide range of nitroaromatic compounds, including those with electron-donating and –withdrawing compounds, as well as nitrobenzene. Notably, the catalyst not only reduced the nitro group but also induced bond cleavage in certain cases. For example, in the case of 4-nitrophenylpalmitate, the catalyst not only reduced the nitro group but also broke the bond between the –O and –C=O groups, resulting in the formation of 4-AP (Table 1, entry 5). Likewise, 4-nitrobenzaldehyde, 4-nitrobenzoic acid and 4-nitroacetophenone were reduced to 4-aminobenzyl alcohol, 4-aminobenzaldehyde and 4-aminostyrene, respectively (Table 1, entries 12–14). The solid products were confirmed by melting point determination, and the structures of several products were identified through GC–MS analysis (refer to Figure S8–S12 in the Supplementary Information). It should be noted that selectivity of all compounds were 100% because this catalyst could promote the reduction very well and the products of this reaction were single products and did not show any side products during the reaction.

**Table 1:** Reduction reaction of nitroarenes in the present of GO–Cu–ASP–m-Co_3_O_4_ MOF^a^.


Entry	1	2	Time (min:sec)	Conversion (%)
1	4-Nitrophenol	4-Aminophenol	00:57	100
2	4-Nitroaniline	4-Aminoaniline	12:00	100
3	2-Nitroaniline	2-Aminoaniline	26:00	100
4	Nitrobenzene	Aminobenzene	18:00	100
5	4-Nitrophenyl palmitate	4-Aminophenol	00:40	100
6^b^	2-Nitrochlorobenzene	2-Aminochlorobenzene	120:00	100
7^b^	4-Nitrophenylhydrazine	4-Aminophenylhydrazine	10:00	100
8^b^	2,4-Dinitrophenylhydrazin	2,4-Diaminophenylhydrazin	35:00	100
9^b^	1-Bromo-2-nitrobenzene	1-Bromo-2-aminobenzene	100:00	100
10^b^	4-Nitrobenzyl bromide	4-Aminobenzyl bromide	40:00	100
11^b^	1,3-Dinitrobenzene	1,3-Diaminobenzene	22:00	100
12	4-Nitrobenzaldehyde	4-Aminobenzyl alcohol	04:00	100
13	4-Nitrobenzoic acid	4-Amino benzaldehyde	01:00	100
14	4-Nitroacetophenone	4-aminostyrene	00:30	100
15^b^	3,5-Dinitrobenzoic acid	3,5-Diaminobenzoic acid	03:00	100

^a^Condition: *p*-nitroarene (0.5 mmol), Catalyst (30 mg), NaBH_4_ (7.5 mmol) and H_2_O at r.t

^b^EtOH/H_2_O (1:3).

Based on the mechanism proposed in recent studies on 4-NP reduction^80,81^, a catalyst can provide an active surface for catalytic reduction. Upon addition of NaBH_4_, 4-NP is protonated and forms 4-nitrophenolate as an intermediate. The reduction process commences with the addition of GO–Cu–ASP–m-Co_3_O_4_ MOF and the transfer of hydride from NaBH_4_ to 4-NP. Once all the oxygen in the system is consumed, the reduction of 4-NP commences. The distribution and accumulation of hydrogen gas bubbles on the catalyst and within the reaction environment create favorable conditions for catalytic reduction. Subsequently, a water molecule is released from the catalyst surface, allowing for the release of 4-AP and initiating the next catalytic cycle. The proposed mechanism for this reaction is represented in Figure S13.

The proposed mechanism, as well as experimental results, unequivocally demonstrate the indispensable role of a reducing agent, in conjunction with a catalyst containing an active metal, in facilitating the reaction. To investigate the influential factors affecting the progress of the 4-NP reduction process, a model reaction was conducted using controlled catalysts at various stages of the catalyst synthesis (Table S1). The control samples exhibited no observable color changes or product formation, underscoring the necessity of an active site, particularly a metal, for effective catalysis (Table S1, entries 13–17 and Figure S6). For instance, GO is unable to promote the reaction as it lacks any active sites for reaction advancement, while, the presence of copper metal is highly suitable for the reduction process and enhances the reaction's yield up to 35%. Additionally, the inclusion of cobalt (Co) nanoparticles, in addition to copper (Cu), had a pronounced effect on the reaction progress, resulting in an efficiency exceeding 50%, which can be attributed to the synergistic effect of two metals. Notably, the most favorable outcomes were obtained with the final catalyst, owing to synergistic effect of Cu, Co, and GO within the MOF structure. The UV–Vis spectra of these processes in the presence of the catalyst are presented in Figure S6.

**Figure 5:**
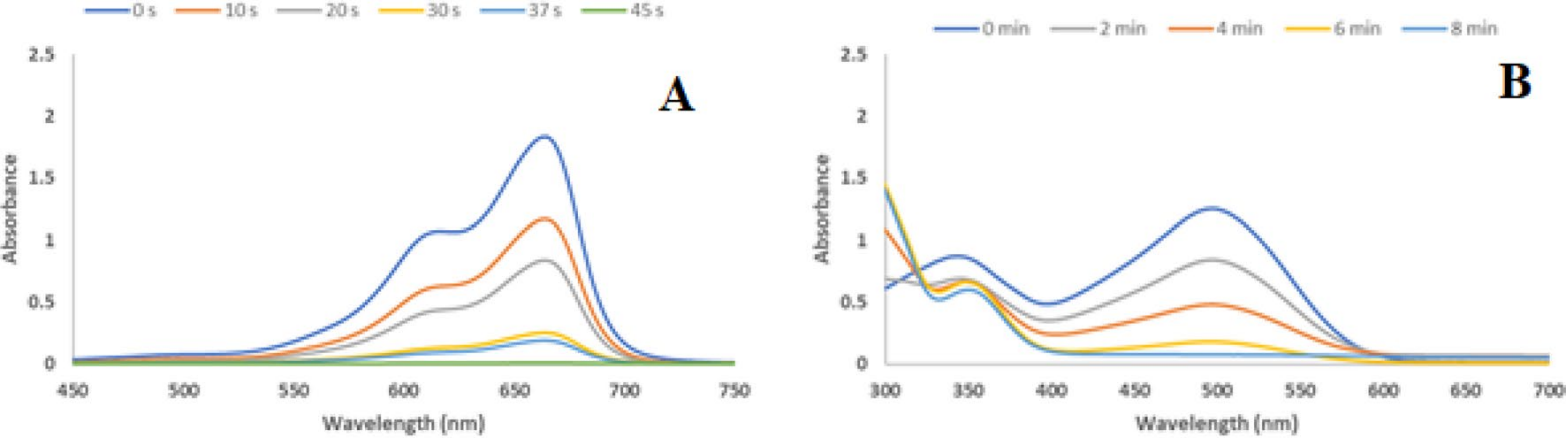
Time-dependent UV–Vis spectra for the reduction of (**A**) MB and (**B**) CR with NaBH_4_ catalyzed by GO–Cu–ASP–m-Co_3_O_4_ MOF.

Furthermore, the reduction of CR and MB using GO–Cu–ASP–m-Co_3_O_4_ MOF was investigated. The reduction of CR and MB only occurred by NaBH_4_ and GO–Cu–ASP–m-Co_3_O_4_ MOF under ambient conditions, as monitored by UV–Vis. As depicted in Table 2 and Fig. 5, the reduction of CR and MB resulted in breakdown of CR into biphenyl, sodium (4-amino-1-naphthalene)-sulfonate, and nitrogen as well as the formation of LMB (leucomethylene blue). The absorption peaks of CR and MB at 493 and 344 nm and 664 nm, respectively, reduced over time, indicating the progress of the reduction procedure (Fig. 5A,B). The reduction of MB was completed within 45 s, with both peaks completely disappearing and the solution changing from dark blue to colorless. Without any catalysts, no change occurred, and the solution remained dark blue. However, the presence of Cu and Co nanoparticles significantly accelerated the reaction, facilitating the rapid absorption of MB from the solution (Fig. 5A). Similarly, at the onset of the CR reduction process in the presence of GO–Cu–ASP–m-Co_3_O_4_ MOF, two absorption bands were observed at 350 and 498 nm. Gradually, both peaks diminished, and after 8 min, they were completely eliminated, attended by a color change from dark red to colorless. Conversely, this reaction exhibited no changes without catalyst, with the solution remaining dark red. However, with the presence of Cu and Co nanoparticles, significant progress was achieved, leading to the absorption of CR from the reaction solution (Fig. 5B).

**Table 2:** Reduction products of MB and CR with GO–Cu–ASP–m-Co_3_O_4_ MOF^a^.


Entry	Conversion (%)	Time (min:sec)
a	100	00:45
b	100	08:00

^a^Reaction condition: *MB and CR* (0.5 mmol), Catalyst (30 mg), NaBH_4_ (10 mmol) and H_2_O at r.t.

Moving on to compare the performance of GO–Cu–ASP–m-Co_3_O_4_ MOF with other catalysts, recent reports on the reduction of 4-NP, MB, and CR were reviewed (Table S2, entries 1–14). As shown in the table, GO–Cu–ASP–m-Co_3_O_4_ MOF achieved higher conversions under more favorable reaction conditions and shorter reaction times. Moreover, GO–Cu–ASP–m-Co_3_O_4_ MOF catalyst demonstrated easy separation with high stability.

Finally, the recyclability of GO–Cu–ASP–m-Co_3_O_4_ MOF in reducing 4-NP was investigated. To this purpose, after each run GO–Cu–ASP–m-Co_3_O_4_ MOF was magnetically separated, washed, dried, and reused in subsequent reactions and prepared for re-uses in similar reactions. As depicted in Figure S14D, the catalyst exhibited recyclability up to eight runs without a significant decrease in conversion. Additionally, SEM/EDX and FTIR also confirmed the structural stability of the recycled catalyst without any noticeable structural changes (Figures S14A, B and C). To further explore the observed decrease in efficiency, an investigation into the leaching of Cu and Co elements was conducted following the third (0.0003 mmol g^−1^ and 0.012 mmol g^−1^) and final (0.0024 mmol g^−1^ and 0.086 mmol g^−1^) recoveries, respectively. The findings indicate that while a minor reduction in efficiency may be anticipated due to the presence of residual materials within the catalyst structure, leading to the obstruction of specific active sites. Consequently, this reduction in reaction efficiency may be attributed to the entrapment of copper species, consequently blocking the active sites.

## Experimental section

### Materials and instruments

The experimental section provides details on the materials and instruments used, including the chemicals and reagents, as well as the characterization techniques employed to analyze the structure of the GO–Cu–ASP–m-Co_3_O_4_ MOF. All chemicals and reagents, including, Almond peel, graphite powder, H_2_SO_4_, H_3_PO_4_, NaNO_3_, H_2_O_2_ (30%), NH_3_.H_2_O, NaBH_4_, TEOS, MB, CR, asparagine, triethylamine, nitro compounds, cobalt(II)nitrate, copper(II)acetate, toluene, methanol, ethanol, and deionized water, were analytical grade reagents purchased from Sigma-Aldrich and used without further purification. The progress of organic reactions was monitored using thin-layer chromatography (TLC) on aluminum plates coated with silica gel 60 F254 and UV–Vis spectroscopy was performed using an Analytik Jena Specord S 600 BU. GC–MS analysis was conducted using an N/5973N6890A Agilent Technologies instrument. The melting point of solid compounds was determined using an electrothermal 9100 apparatus with open capillaries. After the synthesis of the GO–Cu–ASP–m-Co_3_O_4_ MOF, its structure was characterized using various techniques, including Raman spectroscopy, XRD, TGA, FTIR, VSM, SEM/EDX, BET, HRTEM, and ICP-OES. Raman analyses were performed using a Teksan-N1-541 Spectrum with a wavelength of k = 532 nm. X-ray diffraction patterns were obtained using a Siemens D5000.CuKα instrument in the range of 2θ = 5–90° from a sealed tube. The catalyst was subjected to thermogravimetric analysis (TGA) using a heating rate of 10 °C/min up to a temperature of 700 °C under a nitrogen atmosphere. FTIR spectra were obtained using a PERKIN-ELMER-Spectrum 65 instrument. BET analysis was performed using a BELSORP Mini II instrument, with the samples being degassed at 425 K for 1.5 h. SEM/EDX and HRTEM images were recorded using TESKAN VEGA III LMU and HRTEM FEI Tecnai G^2^ F20 instruments, respectively. VSM analysis was conducted at room temperature using a Vibrating Sample Magnetometer (Model 7300 VSM system, Lake Shore Cryotronic, Inc., Westerville, OH, USA). ICP-OES analysis was performed using a Varian-Vista-pro instrument.

### Synthesis of GO–Cu–ASP–m-Co_3_O_4_ MOF catalyst

#### Preparation of GO (1)

The synthesis of the GO–Cu–ASP–m-Co_3_O_4_ catalyst involved a series of well-defined procedures. Initially, GO was synthesized from graphite using a Hummer's method with slight adjustments^82^. A mixture of graphite (2.5 g) and sodium nitrate (1.25 g) was combined, followed by the addition of concentrated H_2_SO_4_ (54 mL) and H_3_PO_4_ (6 mL) under continuous stirring. Care was taken to maintain the temperature below 15 °C during the gradual addition of KMnO_4_ (7.5 g) to avoid overheating and potential explosion and then it was stirred at 40 °C for 2 h and subsequently diluted with distilled water (69 mL) under continued stirring before being subjected to reflux at 90 °C. After an hour, the reflux was terminated, and cold distilled water (100 mL) was cautiously introduced into the reaction mixture, followed by the gradual addition of H_2_O_2_ (7.5 mL). The resulting mixture underwent thorough washing with water, subsequent centrifugation, and finally drying at 80 °C for 16 h, yielding GO sheets (**1**).

#### Synthesis of GO–Cu (2)

To achieve the synthesis of GO–Cu, a meticulously executed procedure was implemented. Initially, a suspension of GO (3 g) in toluene (60 mL) is prepared and subjected to continuous stirring until a homogeneous state was attained. Subsequently, a solution containing copper(II)acetate in methanol (0.6 g, 10 mL) introduced into the aforementioned suspension, and the mixture underwent reflux for a duration of 18 h. Following this reflux process, a precipitate (**2**) materialized, which was subsequently isolated through filtration, carefully washed by MeOH/toluene, subsequently dried under air.

#### Synthesis of GO–Cu–ASP (3)

In order to accomplish this objective, a solution of GO–Cu (3 g) was dispersed in a combination of hot distilled water (15 mL) and ethanol (35 mL) using ultrasonic irradiation (100 W) for a specified duration. Subsequently, asparagine (9 g) and trimethylamine (2 mL) were subjected into the mixture and dispersed for an additional duration. The new mixture was then subjected to reflux at 75 °C with vigorous mechanical stirring under an atmosphere of argon gas for a period of 24 h. The black precipitate (**3**) was subsequently filtered, washed with ethanol, and dried at a temperature of 45 °C for a duration of 6 h.

#### Plant material and extract preparation of Almond peel

The Almond peel was prepared in April 2022 from the market of Tehran province, Iran and its optimum commercial maturity is available in local market. The voucher specimen has been identified by Sharififar and deposited in the Herbarium Center of Department of Pharmacognosy (KF1234). The collected biomaterial was extensively washed under tap water to remove any particulate sprayed with distilled water and dried.

#### Synthesis of m-Co_3_O_4_ (4)

In order to achieve this objective, a solution of Co(NO_3_)_2_⋅6H_2_O (10 g) in distilled water (100 mL) was prepared. Subsequently, Almond peel biotemplate was carefully softened in a mortar and then immersed in the cobalt solution at a 1:1 ratio, followed by stirring for a duration of 12 h. Upon completion, the biotemplate was collected and subjected to calcination at 350 °C for 2 h, resulting in the synthesis of m-Co_3_O_4_ (**4**).

#### Synthesis of GO–Cu–ASP–m-Co_3_O_4_ MOF (5)

**Figure 6:**
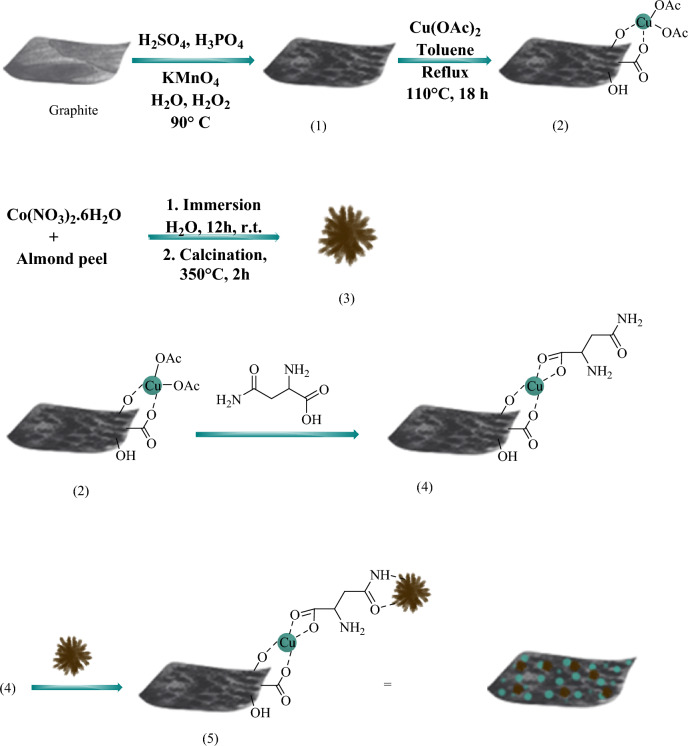
The possible formation process of GO–Cu–ASP–m-Co_3_O_4_ MOF.

GO–Cu–ASP (1 g) was dispersed in distilled water/EtOH (1:1, 50 mL) through the application of ultrasonic irradiation (100 W) for a duration of half the time. Subsequently, m-Co_3_O_4_ (1 g) was introduced to the mixture and dispersed for an additional half of the allotted time. The mixture was then transfered to reflux at 75 °C, while simultaneously undergoing rapid mechanical stirring for a period of 24 h. Following the completion of the reaction, the precipitate (**5**) collected by using a magnet, and subsequently washed with water and ethanol (1:1) three times and then dried in an oven at a temperature of 70 °C for a duration of 12 h. A stepwise depiction of the synthetic process involved in the formation of the GO–Cu–ASP–m-Co_3_O_4_ MOF can be detected in Fig. 6.

#### Typical procedure for the reduction of nitroarenes

A solution having 0.5 mmol of nitroarene in 3 mL of H_2_O was meticulously prepared and combined with a fresh solution of NaBH_4_ (3.75 M in 2 mL H_2_O) and GO–Cu–ASP–m-Co_3_O_4_ MOF (30 mg). The mixture was thoroughly stirred, ensuring homogeneity. The progress of the reaction was monitored at two-minute intervals using UV–Vis spectroscopy (200–800 nm). Following the completion of the reaction, the GO–Cu–ASP–m-Co_3_O_4_ MOF was magnetically isolated, subjected to a water/ethanol wash, dried, and subsequently employed for another cycle under identical conditions. Conversely, the reaction solution was extracted using ethyl acetate, and a diluted solution (25 mL, 0.2 M) was prepared. The purity of the products was ascertained through GC–MS spectroscopy, while the conversion rates were determined utilizing the data obtained from UV–Vis spectroscopy, employing the subsequent equation**:**

$$Percentage conversion= \frac{\left(A\left(initial\right)-A\left(final\right)\right)\times 100\%}{A \left(initial\right)}$$

#### Typical procedure of the reduction of CR and MB

The catalytic activity of GO–Cu–ASP–m-Co_3_O_4_ MOF was further demonstrated through the reduction of CR and MB using NaBH_4_. The experimental conditions for both were identical to those used for nitroarene. Two mixtures were prepared, each containing a solution of CR and MB (0.166 M in 3 mL) along with NaBH_4_ (2 mL, 5 M) and GO–Cu–ASP–m-Co_3_O_4_ MOF (40 mg). Continuous stirring was employed until the colored solutions became colorless, and the reaction progress was monitored using UV–Vis spectroscopy (200–800 nm). Upon completion of the reaction, GO–Cu–ASP–m-Co_3_O_4_ MOF was magnetically isolated, washed with EtOH/water, dried and subsequently utilized for another cycle under the same conditions. On the other hand, the reaction solution was extracted using ethyl acetate, and a diluted solution (10 mL, 0.1 M) was prepared. The purity of the products was determined using GC–MS spectroscopy, and the conversion rates were calculated based on the data obtained from UV–Vis spectroscopy.

### Ethical approval

All applicable international, national, and institutional guidelines for collecting of plant material (Almond peels) were followed.

## Conclusion

In conclusion, a magnetic MOF-based catalyst incorporating mesoporous-Co_3_O_4_ nanoparticles was successfully synthesized and characterized. The GO–Cu–ASP–m-Co_3_O_4_ MOF exhibited exceptional catalytic performance in reduction of nitro compounds, CR and MB under mild conditions. The inclusion of GO–Cu–ASP–m-Co_3_O_4_ MOF significantly enhanced catalytic activity and provided stability to copper and m-Co_3_O_4_ within the catalyst structure, facilitating better recovery. The reduction progress was monitored using UV–Vis spectroscopy, and the conversion rates were determined. The catalyst displayed remarkable recyclability, maintaining relatively stable catalytic activity over eight runs with only minimal decrease in product conversion. These findings contribute significantly to the advancement of efficient methodologies for decolorizing and eliminating pollutants from wastewater.

### Supplementary Information

Supplementary Information.

## Data Availability

All data generated or analyzed during this study are included in this published article [and its supplementary information files].
