# Supplementary material for: Facile Cu–MOF-derived Co3O4 mesoporous-structure as a cooperative catalyst for the reduction nitroarenes and dyes
Source: Sci Rep. 2024 Mar 21;14:6846. doi: 10.1038/s41598-024-52708-x (PMC10958026; doi:10.1038/s41598-024-52708-x)
Supplement: Supplementary file 1 — Supplementary Information. [file 41598_2024_52708_MOESM1_ESM.pdf]

Supporting information for the manuscript

Table S1. Optimization of reaction condition of reduction of 4-NP[a]

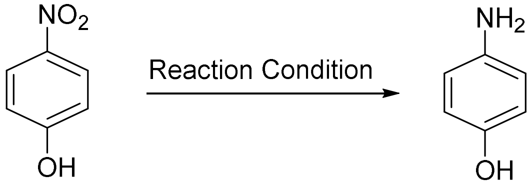

Entry	Catalyst (mg)	$NaBH_4$ (mmol)	Solvent (mL)	Time (min:sec)	Conversion (%)
**1**	GO-Cu-ASP-m-Co_3O_4 MOF (30)	5	5	2:00	100
**2**	**GO-Cu-ASP-m-Co_3O_4 MOF (30)**	**7.5**	**5**	**0:57**	**100**
**3**	GO-Cu-ASP-m-Co_3O_4 MOF (30)	10	5	0:45	100
**4**	GO-Cu-ASP-m-Co_3O_4 MOF (24)	7.5	5	1:40	100
**5**	GO-Cu-ASP-m-Co_3O_4 MOF (40)	7.5	5	0:52	100
**6**	GO-Cu-ASP-m-Co_3O_4 MOF (24)	10	5	0:55	100
**7**	GO-Cu-ASP-m-Co_3O_4 MOF (40)	5	5	1:14	100
**8**	GO-Cu-ASP-m-Co_3O_4 MOF (30)	7.5	2.5	1:06	100
**9**	GO-Cu-ASP-m-Co_3O_4 MOF (16)	7.5	5	1:41	100
**10**	GO-Cu-ASP-m-Co_3O_4 MOF (8)	7.5	5	1:49	100
**11**	-	7.5	5	180:00	Trace
**12**	GO-Cu-ASP-m-Co_3O_4 MOF (30)	0	5	0:57	30
**13**	GO (30)	7.5	5	0:57	Trace
**14**	GO-Cu (30)	7.5	5	0:57	35
**15**	GO-Cu-ASP (30)	7.5	5	0:57	40
**16**	m-Co_3O_4 (30)	7.5	5	0:57	57
**17**	GO-ASP-m-Co_3O_4 (30)	7.5	5	0:57	70

[a] Reaction condition: 4-NP (0.5 mmol) under different condition.

Table S2. The catalytic activity comparison of this work with previous literatures of catalytic reduction of 4-NP to 4-AP (1-6), reduction of methylene blue (7-10), reduction of congo red (11-14).

Entry	**Catalyst (Amount)**	**Reaction condition Solvent/Reductant/Temperature (°C)**	**Time (min:sec)**	**Conv. (%)**	**[Ref]**
**1**	**GO-Cu-ASP-m-Co_3O_4 MOF (30 mg)**	**H_2O/ $NaBH_4$/ r.t.**	**00:57**	**100**	**TW**
2	g-C_3N_4@Ni_3C (23 mg)	H_2O/ $NaBH_4$/ 25	3:00	98	[81]
3	Au−Co_3O_4 (2 mg)	H_2O/ $NaBH_4$/ r.t.	5:30	99	[82]
4	GMS (1 mg)	H_2O/ $NaBH_4$/ r.t.	10:00	99	[83]
5	MXene@AuNPs20 (1 mg)	H_2O/ $NaBH_4$/ r.t.	33:00	99	[84]
6	AuAS (2 mg)	H_2O/ $NaBH_4$/ r.t.	24:00	100	[85]
**7**	**GO-Cu-ASP-m-Co_3O_4 MOF (30 mg)**	**H_2O/ $NaBH_4$/ r.t.**	**00:45**	**100**	**TW**
8	La-$CuBi_2O_4$ (2 mg)	H_2O/ $NaBH_4$/ r.t.	02:30	99	[86]
9	Ag–Cu/TP (10 mg)	H_2O/ $NaBH_4$/ r.t.	10:00	100	[87]
10	AG-$Co_{۳}O_{۴}$ (۲۰۰ mg)	H_2O/ $NaBH_4$/ r.t.	16:00	100	[88]
**11**	**GO-Cu-ASP-m-Co_3O_4 MOF (30 mg)**	**H_2O/ $NaBH_4$/ r.t.**	**08:00**	**100**	**TW**
12	Au NPs/PANI@mGO (10 mg)	H_2O/ $NaBH_4$/ r.t.	20:00	99	[89]
13	PdNPs (4 mg)	H_2O/ $NaBH_4$/ 30	14:00	95.32	[90]
14	Fe_3O_4@PANI@Au (10 mg)	H_2O/ $NaBH_4$/ 25	20:00	99	[91]

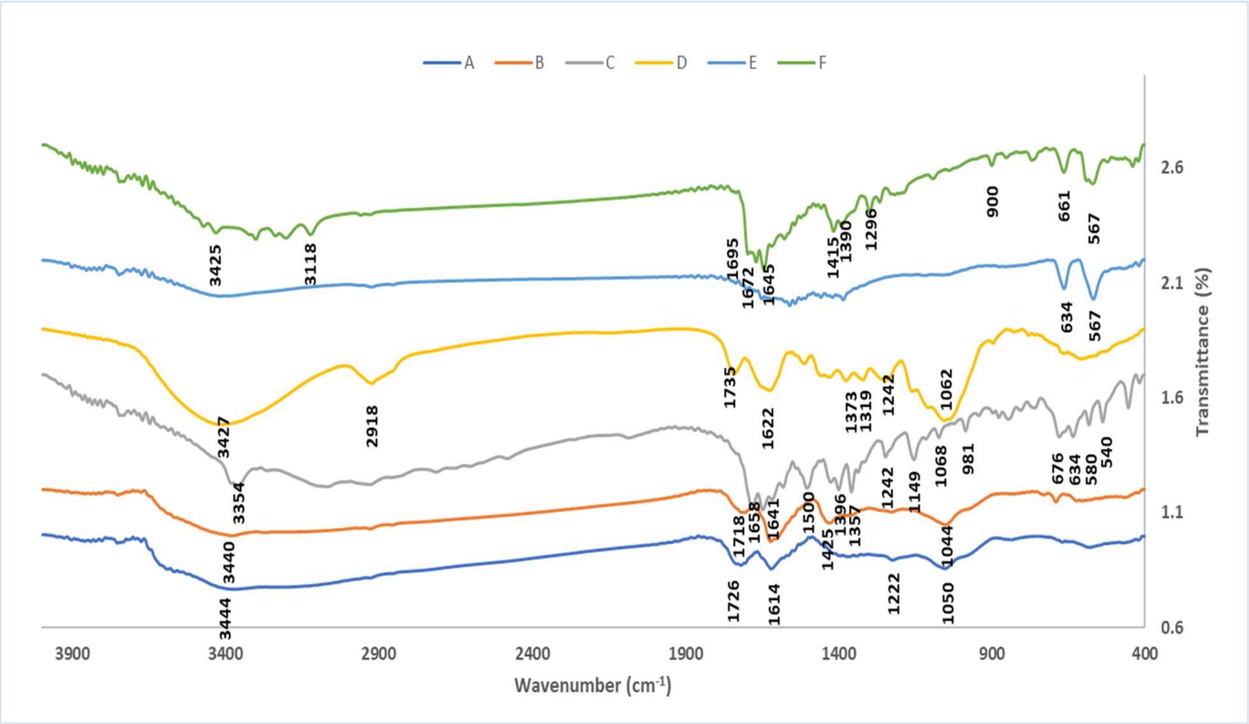

Figure S1. FTIR spectra of GO (A), GO-Cu (B), GO-Cu-ASP (C), Almond peel (D), m-Co_3O_4 (E) and GO-Cu-ASP-m-Co_3O_4 MOF (F).

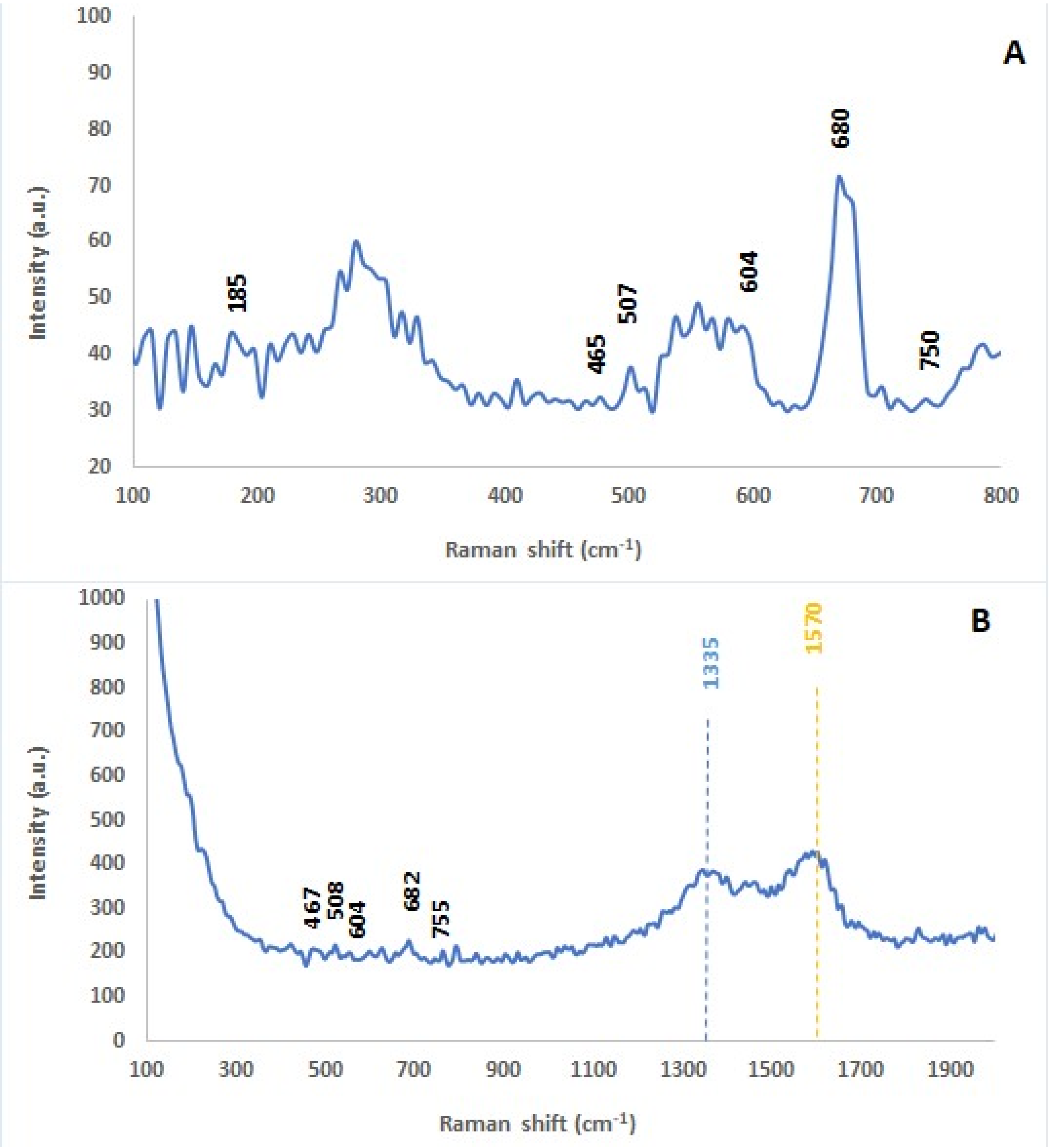

Figure S2. Raman spectra of the (A) m-Co_3O_4 and (B) GO-Cu-ASP-m-Co_3O_4 MOF.

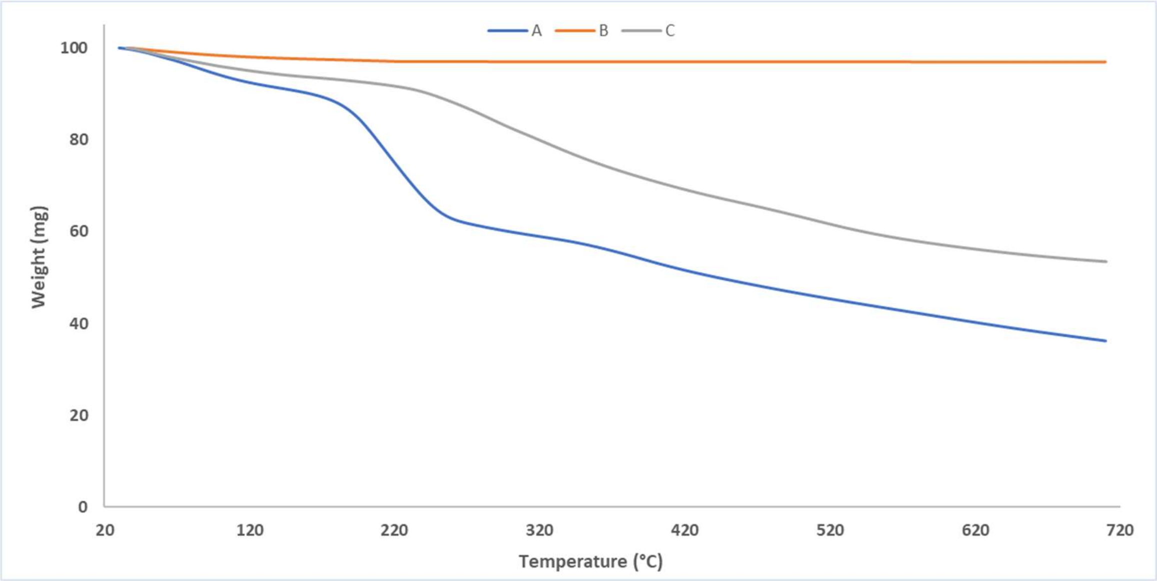

Figure S3. TGA of the (A) GO-Cu, (B) m-Co_3O_4 and (C) GO-Cu-ASP-m-Co_3O_4 MOF.

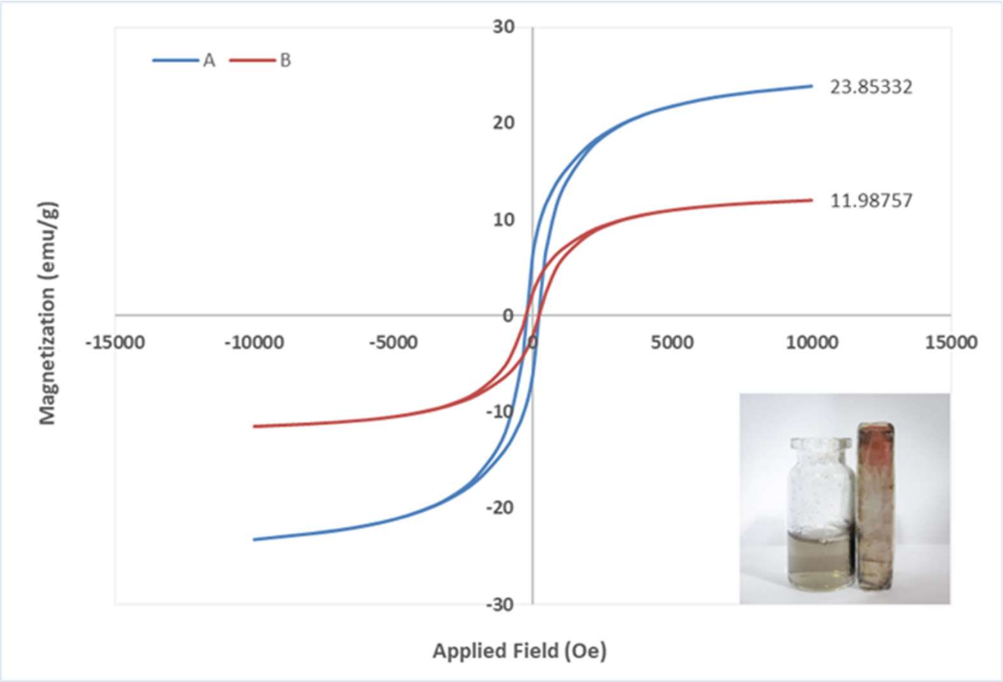

Figure S4. VSM analyses of (A) m-Co_3O_4 and (B) GO-Cu-ASP-m-Co_3O_4 MOF and the picture of reaction mixture after using of a magnetic field.

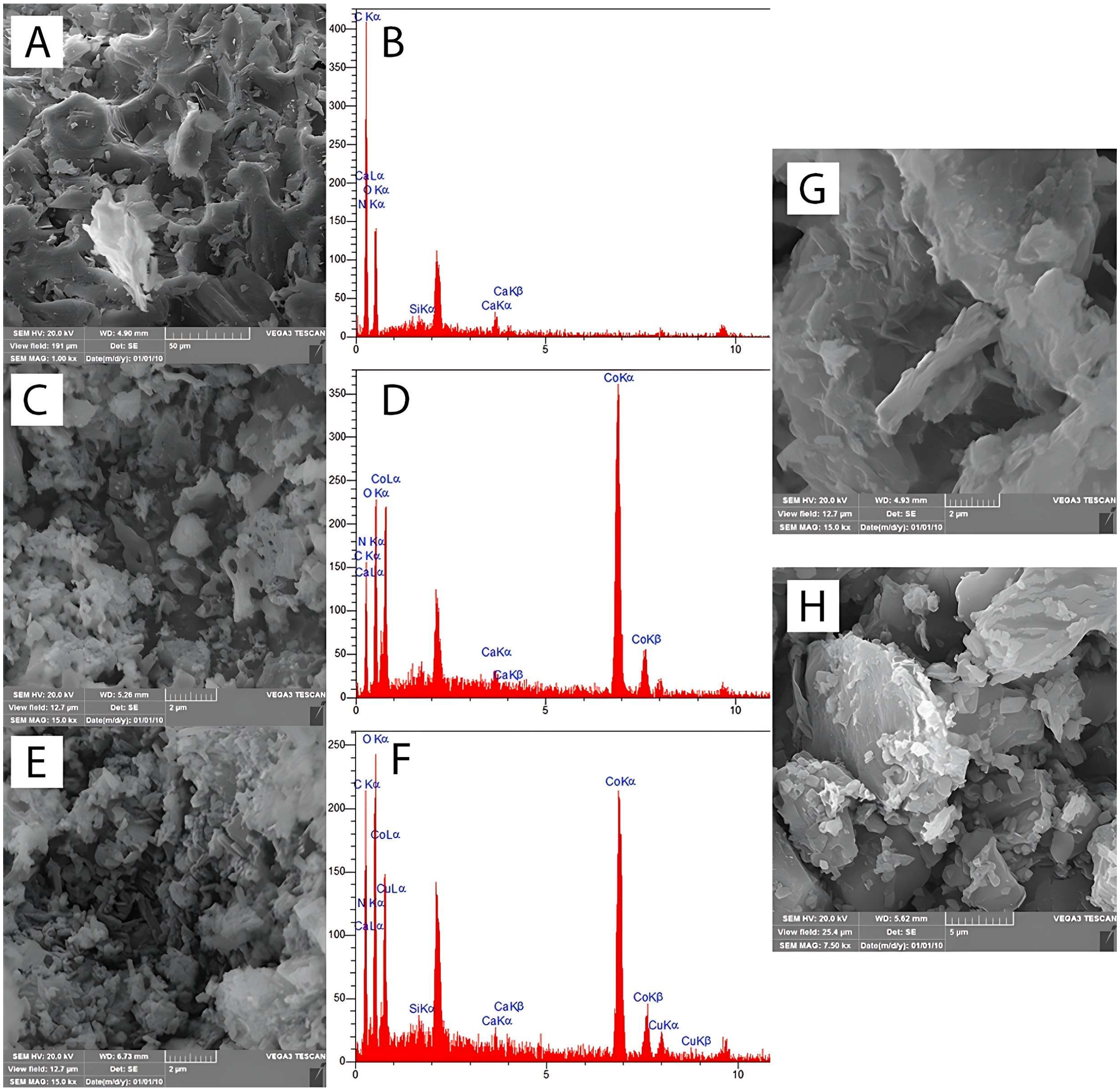

Figure S5. SEM/EDX images of (A,B) Almond peel, (C,D) m-Co_3O_4, (E,F) GO-Cu-ASP-m-Co_3O_4 MOF and SEM images of (G) GO-Cu and (H) GO-Cu-ASP.

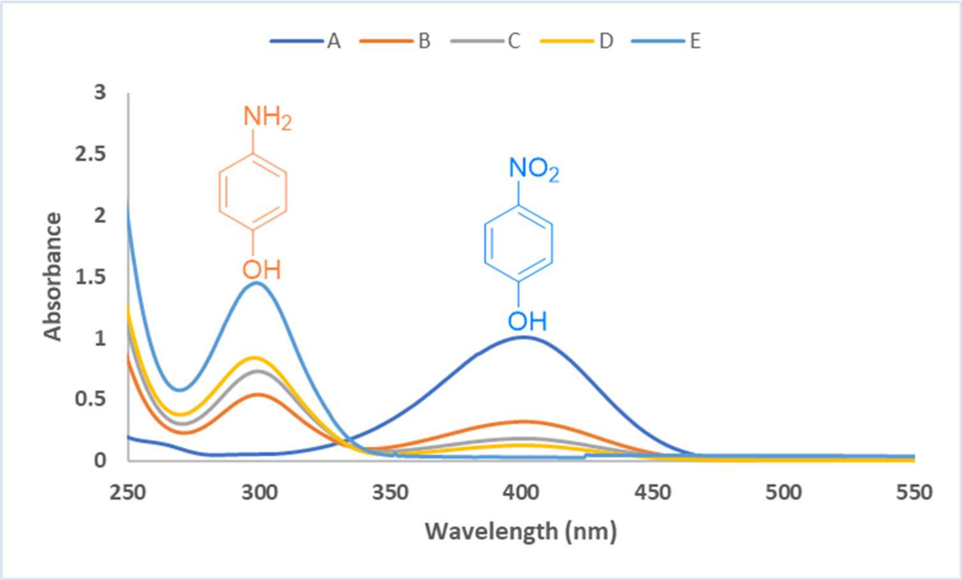

Figure S6. UV-Vis spectra of the (A) GO, (B) GO-Cu, (C) GO-Cu-ASP, (D) m-Co_3O_4, (E) GO-Cu-ASP-m-Co_3O_4 MOF.

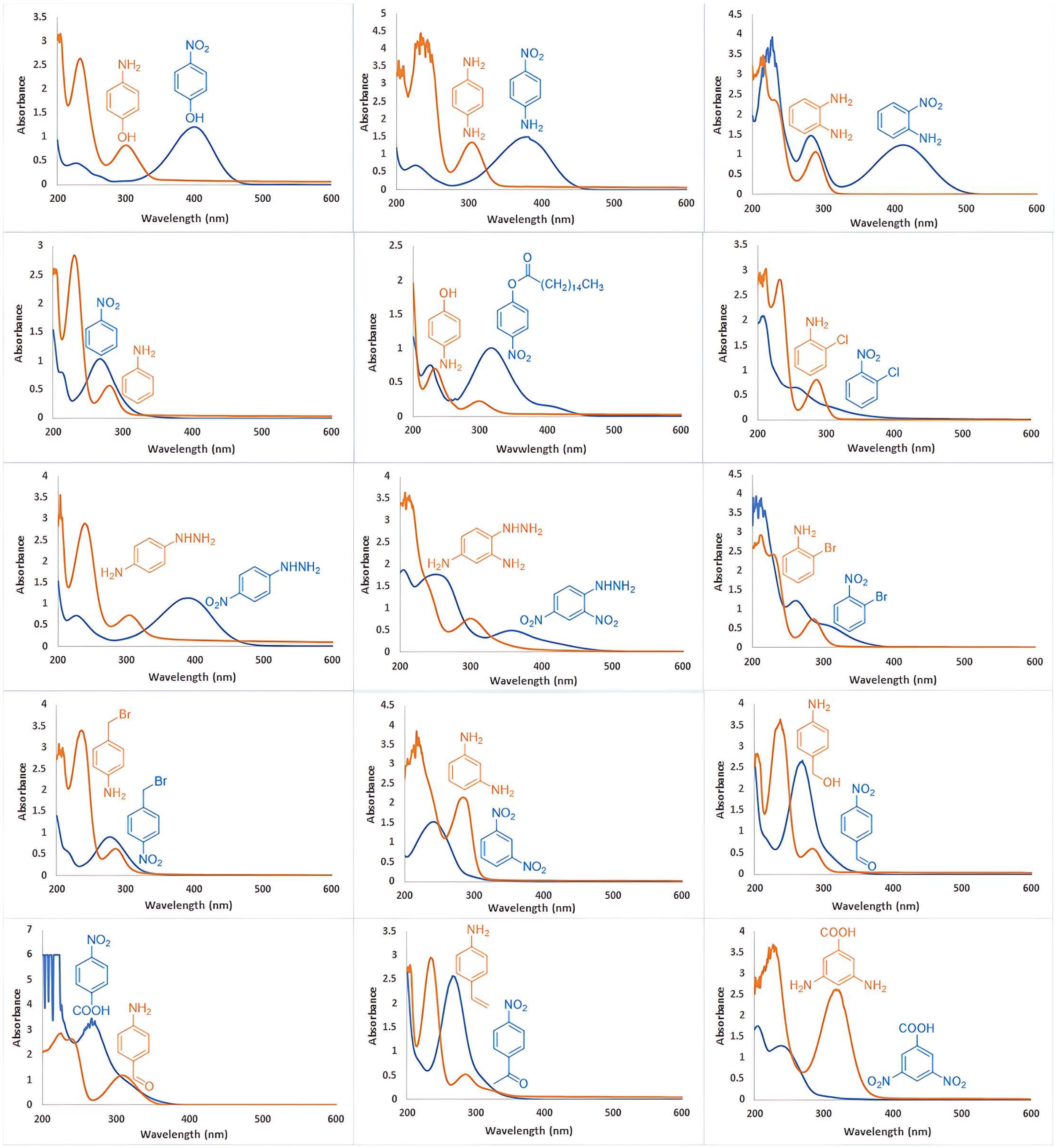

Figure S7. UV-Visible spectra of nitroarene derivatives from reduction reaction catalyzed by GO-Cu-ASP-m-Co_3O_4 MOF.

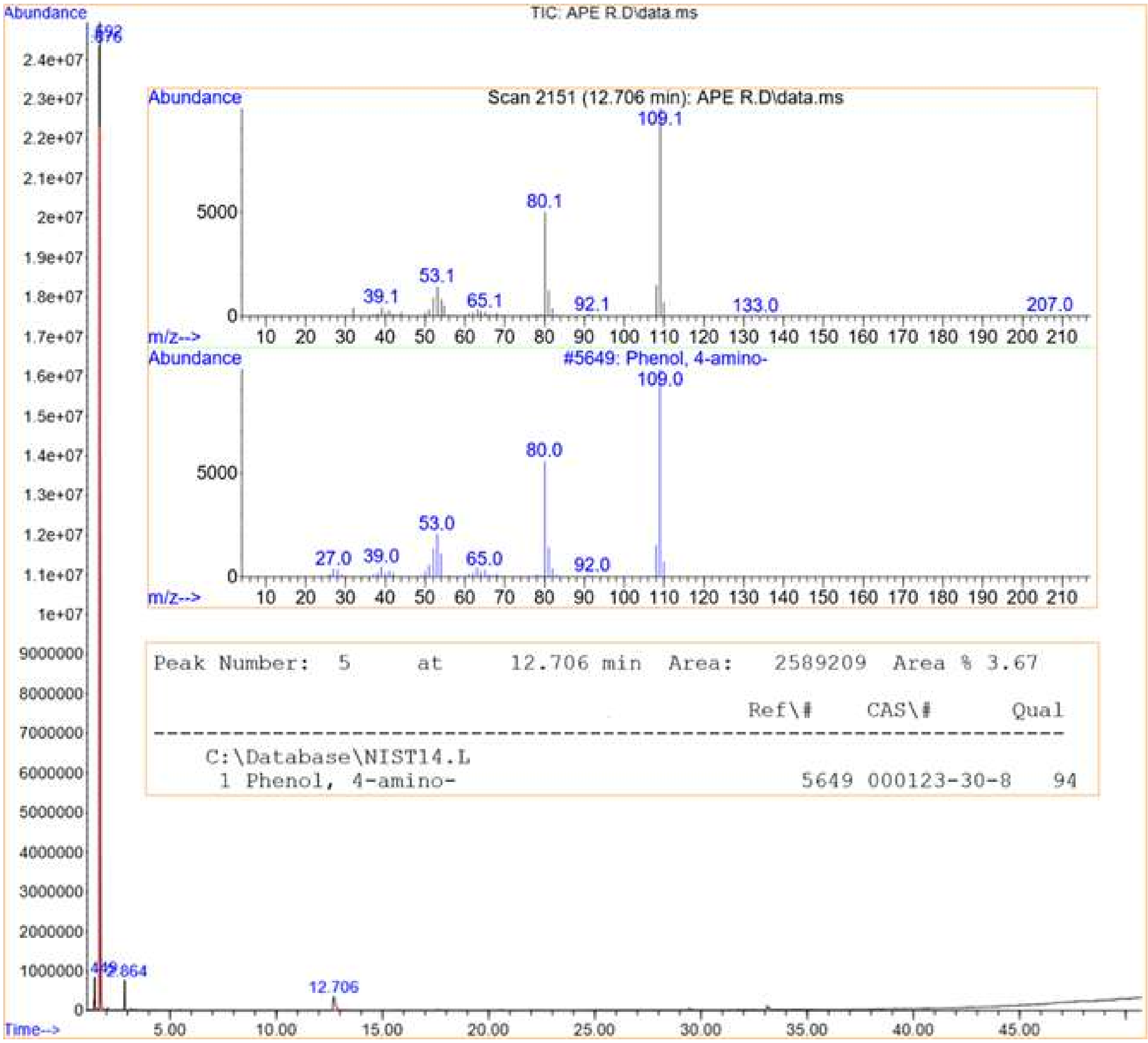

Figure S8. The GC-MS results of 4-AP product after extracting the reaction mixture with ethyl acetate.

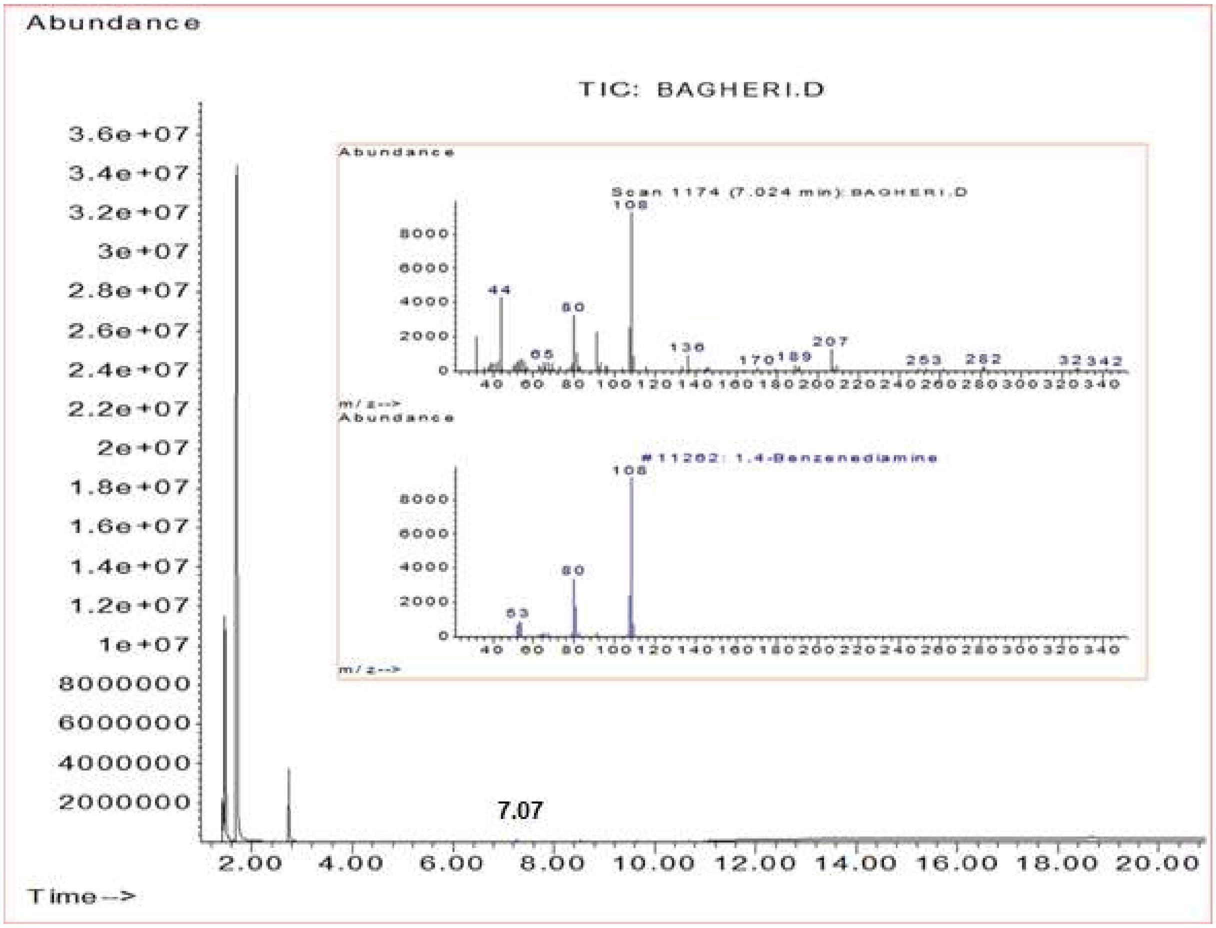

Figure S9. The GC-MS results of 4-Aminoaniline product after extracting the reaction mixture with ethyl acetate

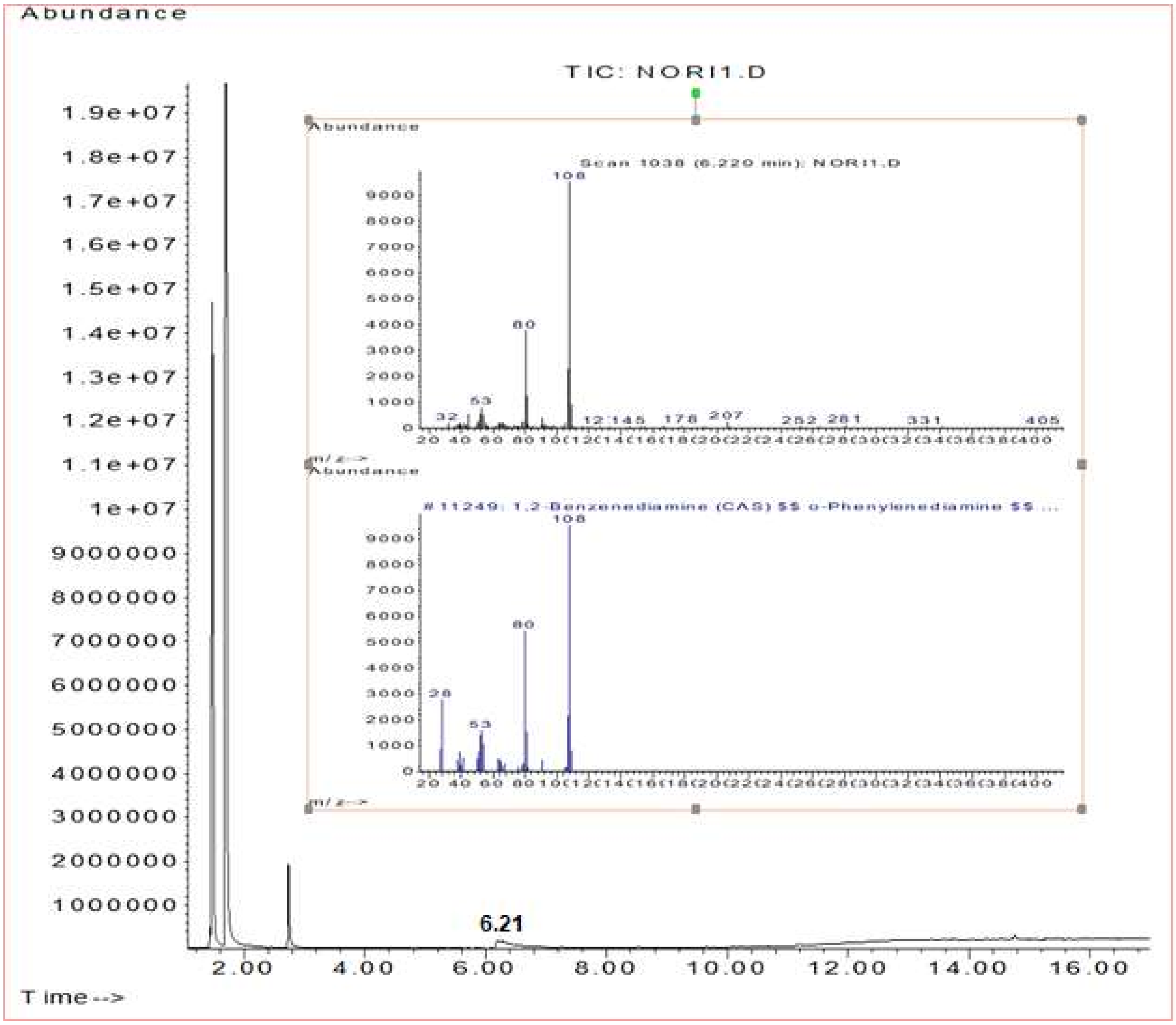

Figure S10. The GC-MS results of 2-Aminoaniline product after extracting the reaction mixture with ethyl acetate

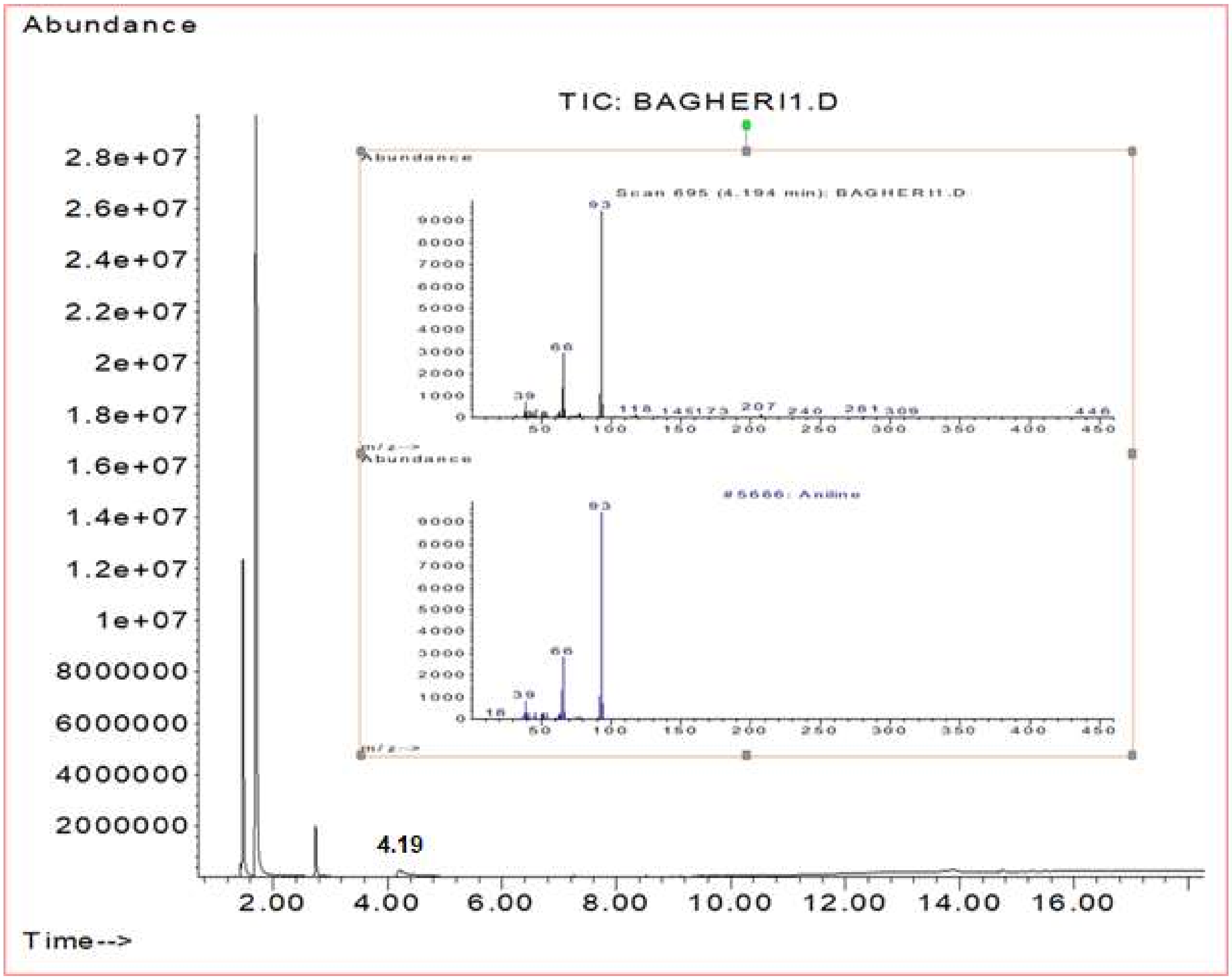

Figure S11. The GC-MS results of Aniline product after extracting the reaction mixture with ethyl acetate

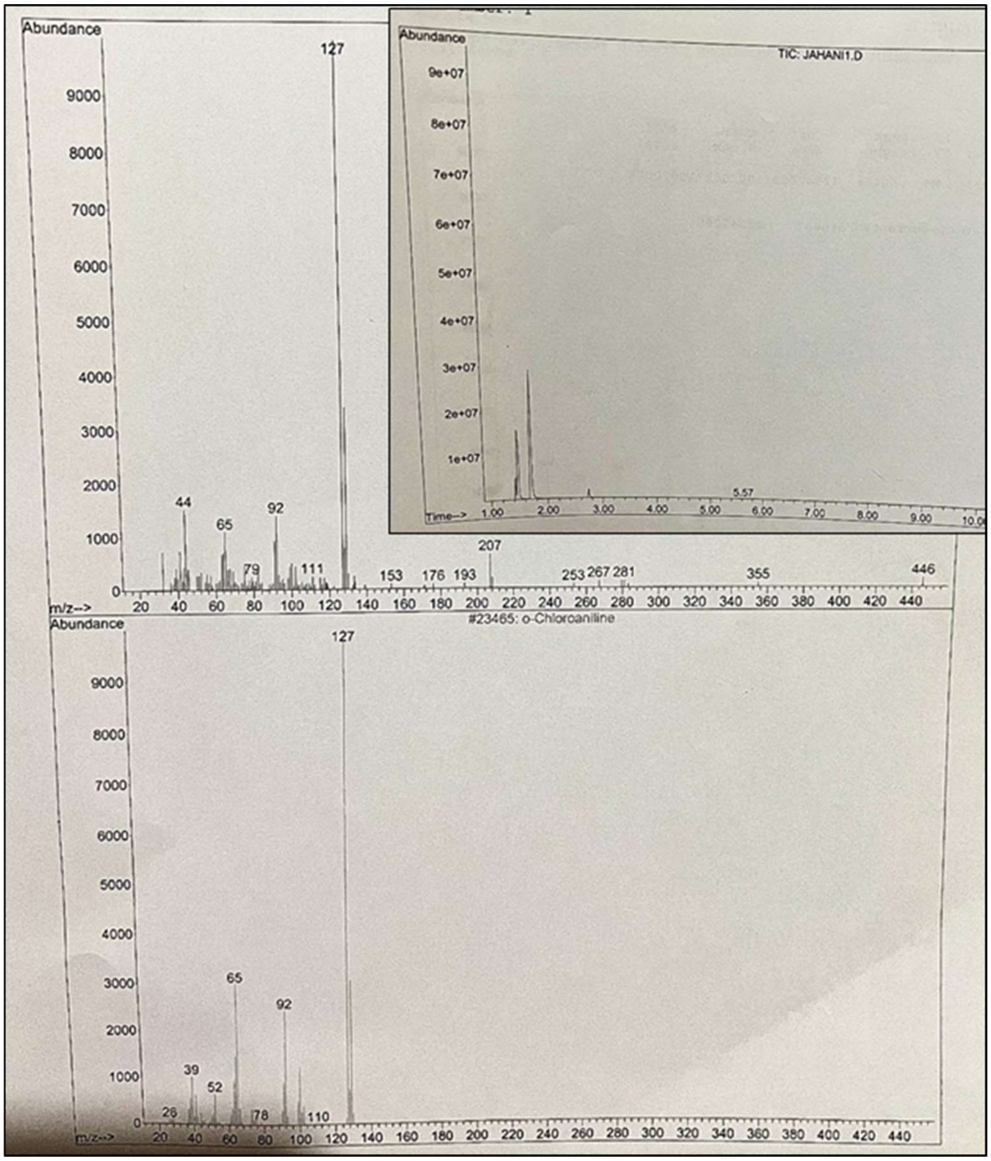

Figure S12. The GC-MS results of 2-Chloroaniline product after extracting the reaction mixture with ethyl acetate.

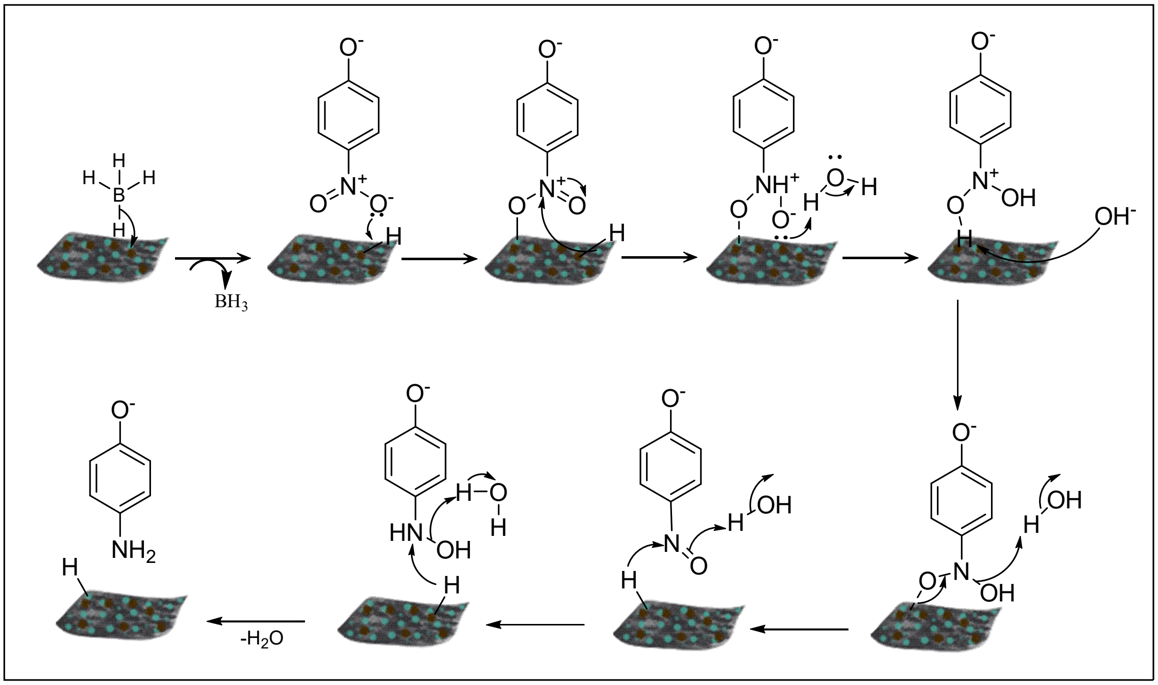

Figure S13. The mechanism of 4-NP reduction by GO-Cu-ASP-m-Co_3O_4 MOF.

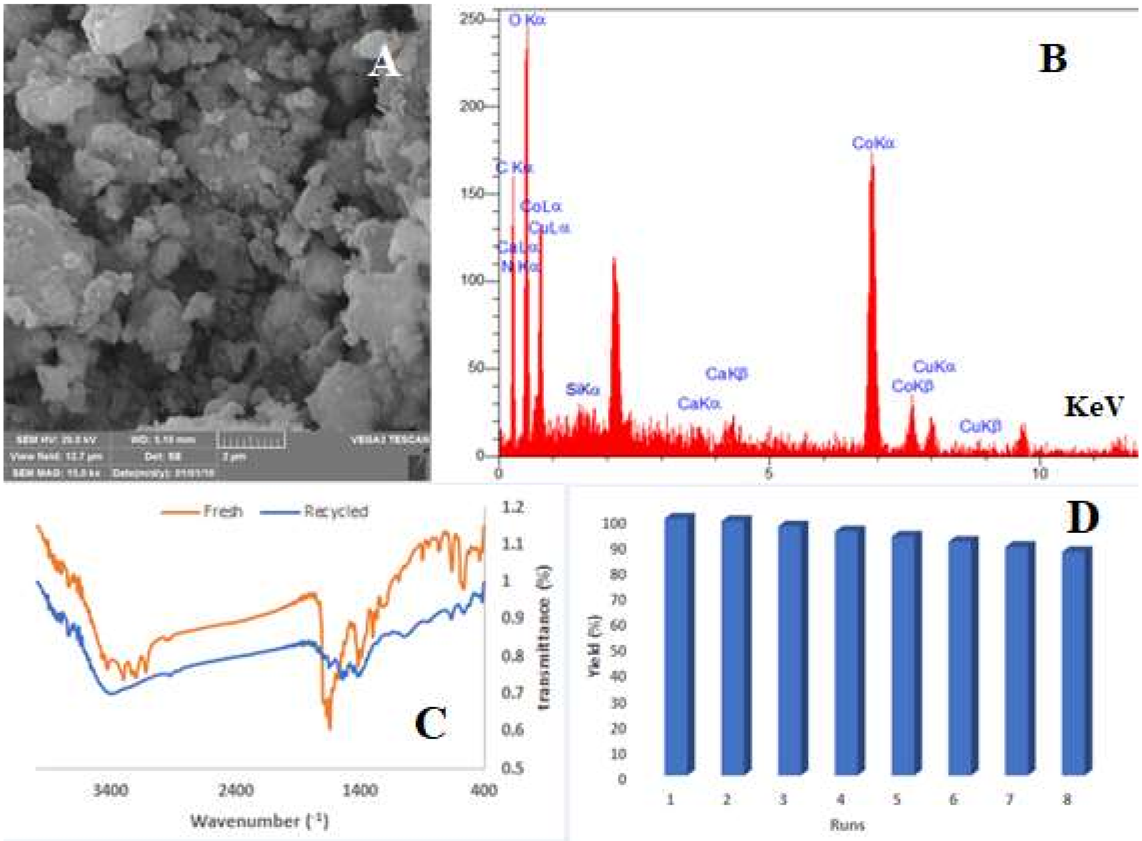

Figure S14. (A,B) SEM/EDX images, (C) FTIR spectra of the fresh and recycled GO-Cu-ASP-m-Co_3O_4 MOF, (D) Recyclability of GO-Cu-ASP-m-Co_3O_4 MOF in reduction of 4-NP.
